# Supplementary material for: Long-Term HBsAg Titer Kinetics with Entecavir/Tenofovir: Implications for Predicting Functional Cure and Low Levels
Source: Diagnostics (Basel). 2024 Feb 25;14(5):495. doi: 10.3390/diagnostics14050495 (PMC10931114; doi:10.3390/diagnostics14050495)
Supplement: Supplementary file 1 [file diagnostics-14-00495-s001.zip › diagnostics-2855497-supplementary.pdf]

Supplementary Materials

Table of contents

Supplementary Figure S1……………………………………………………………2

Supplementary Figure S2……………………………………………………………3

Supplementary Figure S3……………………………………………………………4

Supplementary Table S1……………………………………………………………5

Supplementary Table S2……………………………………………………………7

Supplementary Figure S1. Flow chart of the study.

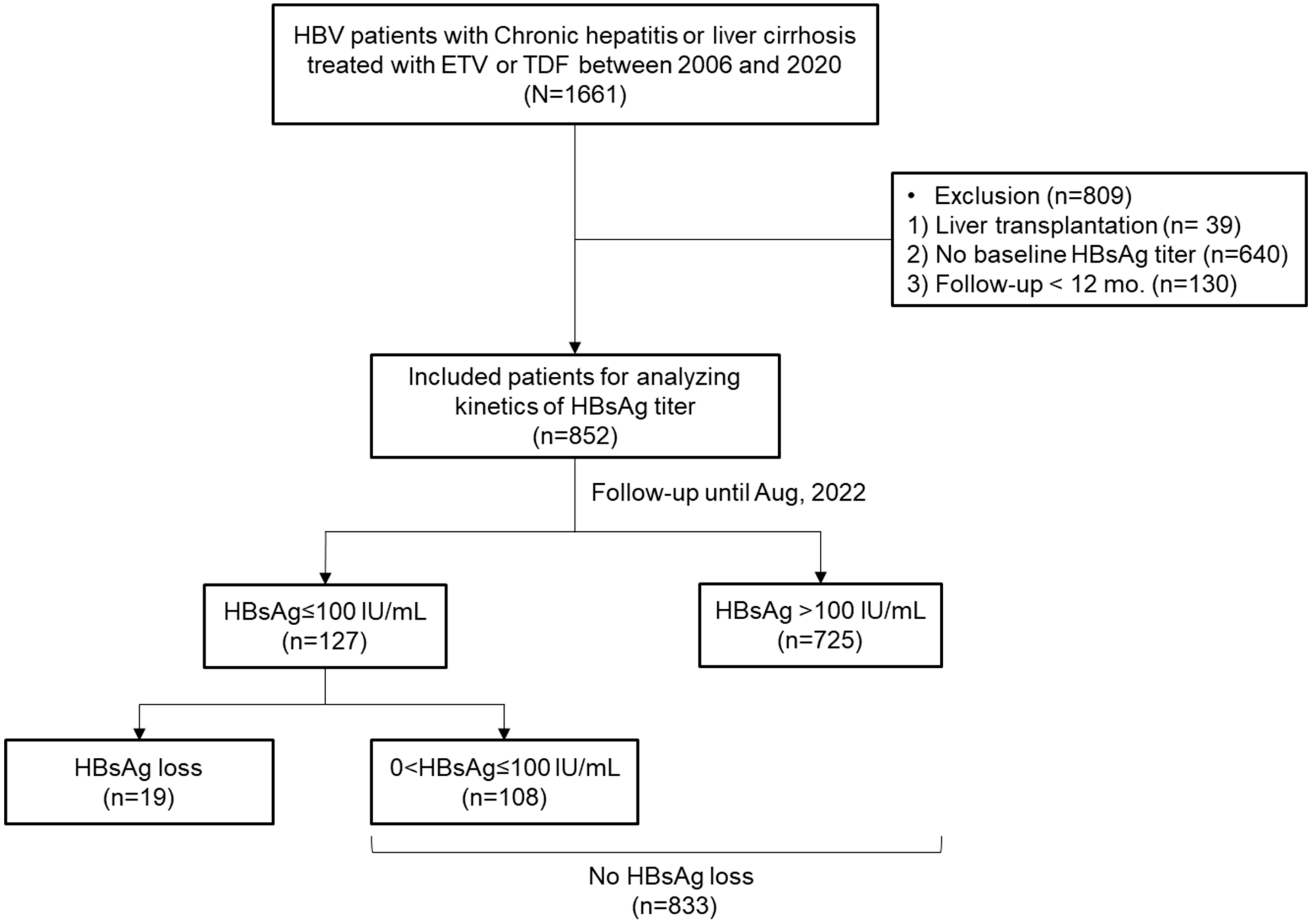

Supplementary Figure S2. Serial changes in the HBsAg level according to the achievement of (A) functional cure and (B) Low HBsAg level after nucleos(t)ide analogue therapy.

(A)

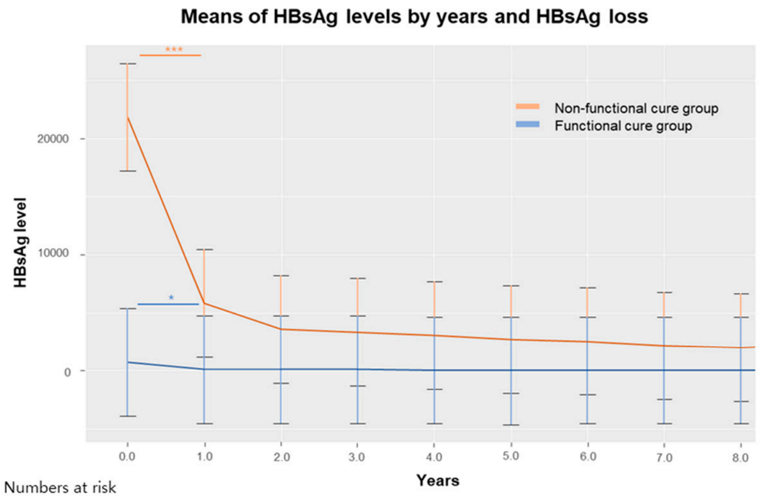

Numbers at risk									
Non-functional cure	833	625	417	269	257	197	238	214	177
Functional cure	19	19	15	10	9	9	8	7	5

(B)

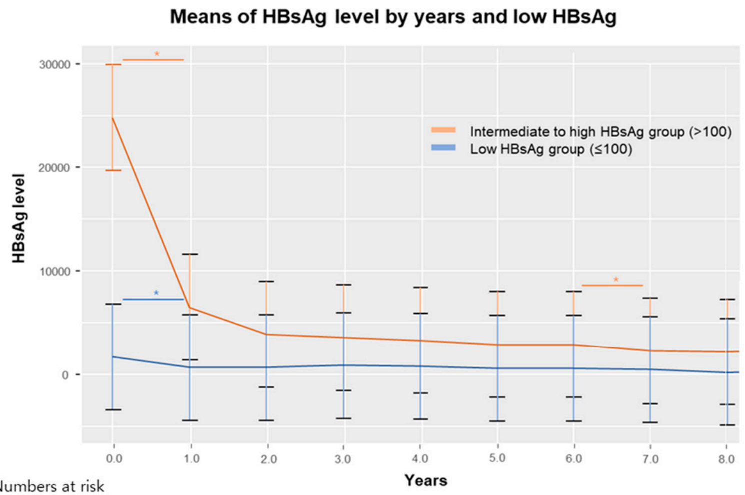

Numbers at risk									
Intermediate to high	725	548	364	233	222	174	200	186	147
Low HBsAg	127	96	68	46	44	32	46	35	35

Supplementary Figure S3. Kaplan-Meier curve for (A) functional cure and (B) low HBsAg level according to type of nucleos(t)ide analogue therapy.

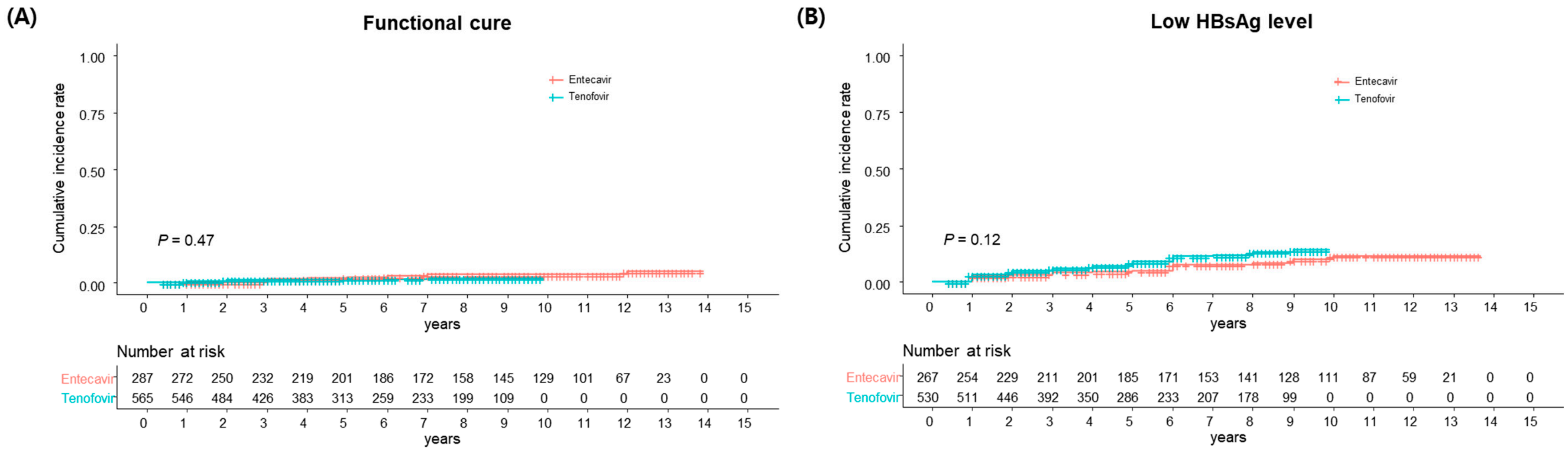

Supplementary Table S1. Baseline characteristics and clinical outcomes of patients achieving HbsAgloss.

No	Age	Sex	Antiviral	HBeAg	Disease status	AST	ALT	T.bil	Alb	Platelet	HBV DNA	Baseline titer	LowHBs _yr	HBsAgloss -Yr	HCC	Progres sion	Regre ssion
1	48	Female	Entecavir	(+) 277.0	Hepatitis	78	73	0.6	3.8	146	6.31	42.34	0.0	3.0	No	No	No
2	48	Female	Entecavir	(+) 1245.8	Hepatitis	128	170	1	3.9	142	6.58	248.29	1.0	3.0	No	No	No
3	61	Female	Entecavir	(+) 1519.4	Hepatitis	44	78	2.5	4.3	218	5.23	72.29	0.0	3.0	No	No	No
4	46	Male	Entecavir	(-)	Hepatitis	253	407	0.9	3.8	184	5.61	1147	2.0	4.0	No	No	No
5	45	Male	Entecavir	(-)	Cirrhosis	287	492	1.7	4.5	136	5.97	78.32	0.0	12.0	No	No	No
6	35	Male	Entecavir	(+) 686.9	Hepatitis	562	683	4.9	3.6	141	6.93	245	3.0	7.0	No	No	No
7	34	Male	Entecavir	(-)	Cirrhosis	94	200	1.2	4.5	159	5.68	47.75	0.0	6.0	No	No	No
8	66	Male	Entecavir	(+)1786.2	Hepatitis	57	62	1.2	4.3	168	8.09	4452.73	1.0	3.0	No	No	No
9	60	Male	Entecavir	(+) 27.9	Cirrhosis	95	153	0.5	4.3	142	6.46	3276	1.0	5.0	No	No	Yes
10	51	Female	Tenofovir	(-)	Cirrhosis	57	89	0.8	4.7	94	3.98	3.64	0.0	7.0	No	No	No
11	59	Male	Tenofovir	(+) 27.9	Cirrhosis	95	153	0.5	4.3	108	6.44	3276.61	1.0	5.0	No	No	Yes
12	65	Female	Tenofovir	(+) 1729.6	Hepatitis	42	52	1.3	4	222	8.04	10.8	0.0	2.0	No	Yes	No
13	69	Male	Tenofovir	(-)	Hepatitis	24	21	0.5	4.5	226	7.14	71.72	0.0	1.0	No	No	No
14	51	Male	Tenofovir	(-)	Hepatitis	946	1616	2.6	4	158	6.36	46.85	0.0	2.0	No	No	No
15	65	Female	Tenofovir	(-)	Cirrhosis	23	13	0.7	4.3	68	2.56	0.02	0.0	2.0	No	No	No

16	31	Male	Tenofovir	(+) 19.3	Hepatitis	2201	1987	9.9	4.7	215	3.63	218.55	1.0	1.0	No	No	No
17	55	Male	Tenofovir	(-)	Cirrhosis	1768	3649	11.3	3.9	199	6.02	2.35	0.0	1.0	No	No	Yes
18	60	Male	Tenofovir	(+) 2.9	Hepatitis	40	40	1.2	4.5	165	7.14	35.09	0.0	2.0	No	No	No
19	67	Male	Tenofovir	(-)	Cirrhosis	36	59	1.4	4.9	160	1.67	9.28	0.0	1.0	No	No	No

Supplementary Table S2. Univariate and multivariate Cox regression analysis for functional cure and low HBsAg level in 5-year landmark analysis.

Functional cure

Variables	Univariate analysis			Multivariable analysis		
	HR[1]	95% CI[1]	*P*-value	HR[1]	95% CI[1]	*P* -value
Age	1.06	1.01, 1.11	0.024	1.06	1.01,1.12	0.023
Male	1.37	0.47, 4.01	0.6			
HBeAg (+)	1.01	0.36, 2.83	>0.9			
HBVDNA	1.00	1.00, 1.00	0.3			
Alb	3.51	1.02, 12.2	0.047	6.61	1.31, 33.4	0.022
Plt	1.00	1.00, 1.01	0.4			
AST	1.00	1.00, 1.00	0.001	1.00	1.00, 1.00	0.4
ALT	1.00	1.00, 1.00	<0.001	1.00	1.00, 1.00	0.2
Tbil	1.06	0.96, 1.16	0.3			
INR	0.21	0.01, 6.72	0.4			
Cr	1.04	0.51, 2.09	>0.9			

Low HBsAg level (<2log IU/mL)

Variables	Univariate analysis			Multivariable analysis		
	HR[1]	95% CI[1]	*P* -value	HR[1]	95% CI[1]	*P* -value
Age	1.03	1.01, 1.06	0.013	1.01	0.98, 1.04	0.50
Male	2.04	1.09, 3.83	0.027	1.85	1.00, 3.40	0.048
HBeAg (+)	0.57	0.33, 0.98	0.043	0.88	0.50, 1.54	0.60
HBVDNA	1.00	1.00, 1.00	0.040	1.00	1.00, 1.00	0.10
Alb	0.82	0.53, 1.26	0.40	0.66	0.41, 1.07	0.095
Plt	1.00	1.00, 1.00	0.8			
AST	1.00	1.00, 1.00	0.060	1.00	1.00, 1.00	>0.9
ALT	1.00	1.00, 1.00	0.13	1.00	1.00, 1.00	0.30
Tbil	1.05	0.99, 1.11	0.10	0.99	0.87, 1.11	0.80
INR	1.16	0.70, 1.92	0.60			
Cr	0.95	0.57, 1.56	0.80			

**MELD**	1.03	0.95, 1.13	0.4			
**Antiviral**	0.81	0.28, 1.61	0.7			
**Cirrhosis**	0.55	0.19, 1.61	0.3			
**HBsAg≤10^3 at baseline**	11.1	3.54, 34.9	<0.001	10.3	3.09, 34.6	<0.001
**HBsAg reduction at 1yr**	2.40	0.68, 8.50	0.20	2.96	0.79, 11.1	0.11

**MELD**	1.03	0.99, 1.08	0.20	0.98	0.88, 1.10	0.80
**Antiviral**	1.73	0.91, 3.31	0.10	1.11	0.62, 1.96	0.70
**Cirrhosis**	0.93	0.54, 1.62	0.80			
**HBsAg≤10^3 at baseline***	7.20	4.15, 12.5	<0.001	6.31	3.66, 10.9	<0.001
**HBsAg reduction at 1yr**	2.79	1.30, 5.97	0.008	2.79	1.52, 5.14	<0.001

[1] HR = Hazard Ratio, CI = Confidence Interval, * HBsAg≤100 IU/mL at baseline were excluded
